# Genome-Wide Analysis of the GRAS Gene Family in Barley (*Hordeum vulgare* L.)

**DOI:** 10.3390/genes11050553

**Published:** 2020-05-14

**Authors:** Vinh-Trieu To, Qi Shi, Yueya Zhang, Jin Shi, Chaoqun Shen, Dabing Zhang, Wenguo Cai

**Affiliations:** 1 Joint International Research Laboratory of Metabolic and Developmental Sciences, State Key Laboratory of Hybrid Rice, School of Life Sciences and Biotechnology, Shanghai Jiao Tong University, Shanghai 200240, China; tvinhtrieu@gmail.com (V.-T.T.); 18395205965@163.com (Q.S.); tulipazhyy@163.com (Y.Z.); vervesj@gmail.com (J.S.); chaoqunshen1213@gmail.com (C.S.); zhangdb@sjtu.edu.cn (D.Z.); 2 College of Life Science, Ningxia University, Yinchuan 750021, China; 3 Key Laboratory of Ministry of Education for Conservation and Utilization of Special Biological Resources in Western China, Ningxia University, Yinchuan 750021, China; 4 School of Agriculture, Food and Wine, University of Adelaide, Urrbrae, Adelaide 5064, Australia; 5 Flow Station of Post-Doctoral Scientific Research, School of Life Sciences and Biotechnology, Shanghai Jiao Tong University, Shanghai 200240, China

**Keywords:** *GRAS* gene family, spike, genome-wide analysis, characterization

## Abstract

The GRAS (named after first three identified proteins within this family, GAI, RGA, and SCR) family contains plant-specific genes encoding transcriptional regulators that play a key role in gibberellin (GA) signaling, which regulates plant growth and development. Even though *GRAS* genes have been characterized in some plant species, little research is known about the *GRAS* genes in barley (*Hordeum vulgare* L.). In this study, we observed 62 GRAS members from barley genome, which were grouped into 12 subgroups by using phylogenomic analysis together with the GRAS genes from Arabidopsis (*Arabidopsis thaliana*), maize (*Zea mays*), and rice (*Oryza sativa)*. Chromosome localization and gene structure analysis suggested that duplication events and abundant presence of intronless genes might account for the massive expansion of *GRAS* gene family in barley. The analysis of RNA-seq data indicates the expression pattern of *GRAS* genes in various tissues at different stages in barley. Noteworthy, our qRT-PCR analysis showed the expression of 18 candidate *GRAS* genes abundantly in the developing inflorescence, indicating their potential roles in the barley inflorescence development and reproduction. Collectively, our evolutionary and expression analysis of GRAS family are useful for future functional characterization of GA signaling in barley and agricultural improvement.

## 1. Introduction

Transcription factors bind to the specific *cis*-elements in the promoter region of target genes to modulate their expression. The *GRAS* gene family belongs to plant transcription factors to regulate plant growth and development [1]. The GRAS family is named after the first three-member including Gibberellic Acid Intensive (GAI), Repressor of GAI-3 mutant (RGA), and Scarecrow (SCR) [1]. Generally, proteins in this family consist of a variable N-terminus and a highly conserved C-terminus. The C-terminus is composed of five conserved domains including LHRI (Leucine Heptad Repeat I), VHIID, LHR II (Leucine Heptad Repeat II), PYRE, and SAW [1], while the N-terminus includes a combination of several molecular recognition features, required for protein–protein association or molecular recognition [2]. Furthermore, DELLA and VHYNP domains had been detected in the N-terminus of GRAS proteins in DELLA subfamily, which is a gibberellic acid (GA) signal perception domain [3]. The C-terminal GRAS domain is a transcription repressor-domain regulating gene expression [3]. GRAS-like proteins were also found in bacteria, albeit with methylase activity rather than transcription modification [4]. This implies the origin of plant GRAS from horizontal gene transfer from ancient prokaryote genomes of bacteria.

In the past decades, *GRAS* genes have been identified in a variety of plant species, including 34 in Arabidopsis, 60 in rice, 84 in maize, and 106 in *Populus trichocarpa* [5,6,7]. Previously, GRAS proteins were initially clustered into eight groups, consisting of LS, HAM, PAT1, LISCL, DELLA, SCL3, SHR, and SCR, based on the GRAS members identified from Arabidopsis and rice [5,8]. A phylogenetic tree containing 12 discreet clades was revealed later based on alignment of GRAS protein sequences from a broader range of species including lycophyte, bryophyte, and vascular plants [9]. Nevertheless, there is a slight difference in the subgroupings in recently characterized GRAS families from different species. For instance, the identification and classification of GRAS proteins are divided into 13 subfamilies in *P. trichocarpa* [6], 13 in the tea plant (*Camellia sinensis*) [10], and 16 in *Medicago truncatula* [11]. These studies indicate substantial divergence of GRAS family in flowering plants and more subfamilies may be recognized upon analysis of furthermore plant genomes in the future.

With the advancements of genetics and genomics, the biological functions of some *GRAS* genes family have been investigated. It is known that DELLA proteins (including GAI, RGA, RGL1/2/3 (GAI/RGA-like 1/2/3) in Arabidopsis, SLR1 (Slender1) in rice, and ZmD8 (Dwarf8) in maize) act as master growth inhibitors and key components in GA signaling [12]. DELLA binds to GA activated GID1 (GA-Insensitive Dwarfism1) to form a GA-GID1-DELLA complex, which is subsequently recognized by SCF^SLY1/GID2^ (a Skp, Cullin, F-box containing complex, the F-box protein was named as Sleepy1 (SLY1)/Gibberellin Insensitive Dwarf2 in Arabidopsis) E3 ubiquitin ligase, inducing the proteasomal degradation of DELLA [13,14,15], activating the expression of genes repressed by DELLA protein [16]. Arabidopsis SCL3 is a close paralog of DELLA and functions as GA-positive regulator of root development [17], and SCL3 protein interacts with DELLA protein and inhibits the transcription activity of DELLA to attenuate its own expression [18,19]. Two GRAS proteins belonging to two different subfamilies, SHR and SCR, collectively specify cells as endodermis in the root or bundle sheath in the shoot [20,21]. Similar to that of SHR and SCR, NSP1 and NSP2 belong to two different subfamilies (AtSHR and HAM, respectively, with 20.1% identity) and collectively regulate the nodule development and function in legumes [22,23]. The GA-GID1-DELLA signaling axis is conserved in plants [12].

Remarkably, some GRAS family genes were shown critical for the improvement of many agronomic traits in cereals, such as wheat (*Triticum aestivum*) *Rht1 and Rht2 (Reduced height1 and 2)* and rice MOC1 (*Monoculm 1*) [13,15,24,25]. The functions and working mechanisms of those GRAS family genes are conserved among species, albeit with little variations. For example, tomato (*Lycopersicon esculentum*) LS (Lateral Suppressor), Arabidopsis LAS (*Lateral Suppressor*), and rice MOC1 (*Monoculm 1*) share only about 50% similarity and show a few diversified functions in reproductive stages and controlling axillary meristem initiation. Moreover, they are conserved in promoting shoot branching or tillering [25,26,27]. Importantly, the Arabidopsis *LAS* gene can fully complement the tomato *ls* mutant, suggesting their functional conservation [27]. It is conceivable that detailed characterization of *GRAS* family in cereal crops may be of great application potentials. However, according to our knowledge little is known about the function of *GRAS* family in barley, particularly in the barley inflorescence development.

Barley (*Hordeum vulgare* L.) is the fourth most cultivated cereal crop around the world after rice, maize, and wheat [28], whose grains are mainly used for feeding human, cattle, and brewing beer. The flowering process and the formation of spikelet abundantly contribute to the barley grain yield [29]. With bioinformatics analysis, the published whole genome in barley enables us to understand the functions of GRAS proteins in this crop and inflorescence architectures study. In this paper, 62 *GRAS* genes were identified from the recently released barley genome. We also conducted phylogenetic analysis of these GRAS members and revealed their structural diversity, and showed their diversified expressional patterns, providing a basis for further elucidating the function of barley GRAS.

## 2. Materials and Methods

### 2.1. Identification of GRAS Members in Barley

To find the candidate *GRAS* genes in barley genome, we performed an extensive search and comparisons analysis for GRAS domain in many plant species using HMMER software. The GRAS family information of 34 Arabidopsis, 60 rice, and 86 maize were retrieved from the previous studies (Table S7) [6,7]. The protein sequence of Arabidopsis, rice, and maize downloaded from The Arabidopsis Information Resource (TAIR, https://www.Arabidopsis.org/), Rice Genome Annotation Project (RGAP, http://rice.plantbiology.msu.edu/), and MaizeGDB (http://www.maizegdb.org/, v3) respectively [5,6,7]. The sequence of 176 wheat and 48 *Brachypodium distachyon* GRAS protein sequences were obtained from previous studies [30]. The barley genome (*Hordeum vulgare r1*) was retrieved from the IPK Barley Blast Server (http://webblast.ipk-gatersleben.de/barley_ibsc/) [31,32,33]. Based on the results of searching Hidden Markov Model (HMM) profile of GRAS domain in Arabidopsis, rice, and maize against the barley genome, the relevant information of GRAS domain in barley was extracted using HMMER software (version 3.0, http://hmmer.org). The candidate barley GRAS proteins were selected based on an E-value ≤ e^−10^. To further confirm the GRAS proteins in barley, the data were continually verified by Conserved Domain Database (CDD) in NCBI (https://www.ncbi.nlm.nih.gov/) and Pfam value (the GRAS superfamily, cl15987, GRAS family pfam03514, DELLA family pfam12041) [34]. There is no doubt that numerous proteins were eliminated due to inadequacy, lacking, or anonymous GRAS domains. The composition, physical, and chemical characterization of identified HvGRAS proteins were analyzed using ExPASy software (https://web.expasy.org/protparam/) [35].

### 2.2. Phylogenetic Analysis of GRAS Members

To further explore the evolutionary relationship among six plant species, including Arabidopsis (annual herbaceous dicots), rice (perennial in certain countries and annual cereal grain monocot), barley (major cereal crops), maize, wheat, and *Brachypodium distachyon*, the phylogenetic analysis was constructed. Multiple sequence alignments of GRAS proteins were performed using MEGA software (X version; https://www.megasoftware.net/) with default setting MUSCLE method [36]. Based on this result, the Maximum Likelihood (ML) phylogenetic tree among Arabidopsis, rice, maize, barley, wheat, and *Brachypodium distachyon* was generated by W-IQ-TREE (http://iqtree.cibiv.univie.ac.at/) with default parameters, bootstrap method 1000 [37]. The best-fit substitution model (in our case, nuclear General “Variable time” matrix) was automatically chosen by W-IQ-TREE, followed by tree construction [37]. The evolutionary tree was visualized and modified to circle using the iTOL (https://itol.embl.de/) [38].

### 2.3. Chromosomal Localization and Evolution Pressure of HvGRAS

The chromosomal locations of 62 *HvGRAS* genes were downloaded from the Phyotozome database [31,32,33]. CDS sequences of *HvGRAS* genes were extracted from Phyotozome and aligned using MEGA software (version X; https://www.megasoftware.net/), with Clustal X method—proteins sequence. The results were introduced into DnaSP 6 software to calculate synonymous (Ks) and nonsynonymous (Ka) nucleotide substitution rates of 17 pairs of paralog genes [39]. The possible correlation of gene pair duplication and *HvGRAS* gene location on the barley seven chromosomes was visualized by the Super Circos in TBtools software [40].

### 2.4. Analysis of Conserved Motif and Gene Structure

To investigate the GRAS features domain in barley sequence, multiple sequence alignment of 62 HvGRAS proteins was conducted using Jalview software 2.10.5 version, following the parameters alignment Clustal with defaults and Realign with Clustal [41]. The detailed analysis of conserved motif domains in HvGRAS proteins was performed using the MEME suite tool from the website (http://meme-suite.org/) with non-modification of parameters, except for that the “MEME should find” was 17 [42]. The exon–intron structure of *HvGRAS* was examined using the TBtools and the GFF3 database obtained from Phytozome [33]. The full graphics of conversed motif and gene structure were visualized by TBtools [40]. miRNA target site in barley GRAS family was obtained from the study of overexpression of microRNA171 [43].

### 2.5. Expression Analysis of HvGRAS Members

The expression of *HvGRAS* genes in different tissue was measured using the transcriptome data of RNA sequence from BARLEX (Barley Genome Explorer, https://apex.ipk-gatersleben.de/apex/f?p=284:10) [44]. The expression pattern of spike development was generated from the previous study of the transcriptome profile in barley (Table S4) [45]. The expression value was calculated by the reads per kilobase per million (Table S3). A heatmap of expression pattern profile on log_2_^(FPKM+1)^ scale was analyzed by the TBtools and a hierarchical clustering algorithm to identify the similarity of expression pattern [41].

### 2.6. Plant Materials and Growth

*H. vulgare* cultivar Golden Promise was grown in the chamber at Shanghai Jiao Tong University. The soil mixture was followed by the combination of soil, perlite, and vermiculite (with the ratio of 1:1:0.5). The chamber condition was 16 °C/14 °C—16/8 h day/night period and 50% relative humidity. To investigate the expression level of *HvGRAS* genes in barley inflorescences, the development of barley main shoot apex (MSA) from W2.0 to W6 were collected under the microscope, and the developmental stages were based on the inflorescence architecture study in barley [46,47]. These stages included the double ridge stage (DR), the lemma primordium (LP) stage, the stamen primordium stage (SP), the awn primordium stage (AP), the white anther stage (WA) and the green anther (GrA). Two-week-old seedlings were used as control. At double ridge stages (W2.0), MSAs were collected after sowing 17–20 days, LP stage (W3.0), SP stage (W3.5), AP stage (W4), WA stage (W5-5.5), and GrA stage (W6) [46].

### 2.7. RNA Extraction and Quantitative Reverse Transcription PCR (qRT-PCR)

Total RNA was isolated from tissue by TRIZOL reagent (Invitrogen), followed by the instruction guide in the chemical products. The reserve transcriptional reaction was performed by a PrimeScript RT reagent kit with gDNA Reaser (Takara), followed by the instruction guide in the chemical products. The qPCR was conducted using SYBR Green SuperReal PreMix Plus (TIANGEN) on a CFX96 Real-time PCR machine (Bio-Rad). The internal control in this study was *HvACTIN* (HORVU5Hr1G039850.3) [48]. Three biological replicates with three technical replicates were conducted (Table S6). Detailed primer information is attached to Supplementary Materials (Table S5).

### 2.8. HvGRAS Proteins Interaction Network

To illustrate the protein–protein interaction network in HvGRAS, the orthologs with Arabidopsis were performed to predict the correlation network in barley. Black lines connect the Arabidopsis interacting GRAS proteins based on the protein interaction databases, plant.MAP (http://plants.proteincomplexes.org/), BioGRID (https://thebiogrid.org/), and STRING (http://string-db.org/) [49,50,51]. The HvGRAS protein orthologs were listed in parenthesis. Red color marked genes indicate the HvGRAS expression in spike.

## 3. Results

### 3.1. Identification and Characterization of GRAS Proteins in Barley

Briefly, 62 GRAS proteins identified in barley and designated as HvGRAS3 to HvGRAS62 according to the physical location on the chromosomes, except for HvGRAS1 and HvGRAS2 whose locations on chromosome remained unknown. Furthermore, the basic properties of these GRAS proteins such as molecular weight, the number of amino acid, and theoretical Isoelectric Point (pI) are summarized in Supplementary Table S1. Except for those lacks of certain GRAS domains HvGRAS53 were excluded from this study. The GRAS domain sequence is around 300 amino acids long, whereas HvGRAS53 is relatively short, leading to the unreliable result in phylogeny analysis [10]. The length of GRAS proteins in barley was between 121 and 792 amino acids, and correspondingly the molecular weights were from 30 kDa to 50 kDa (Table S1). The theoretical isoelectric point (pI) value varied from 0.93 (HvGRAS22) to 11.56 (HvGRAS57). Judging from the pI value, more than half of HvGRAS proteins (45/62) were rich acidic amino acid, and the rest HvGRAS proteins (17/62) were alkaline. Almost all HvGRAS proteins belonged to the hydrophilic group because the hydropathicity values were below 0, varying from −0.63 to −0.11. 9 HvGRAS proteins had hydropathicity values above 0, ranging from 0.015 to 0.107. The structure and stability of the HvGRAS proteins were determined based on the instability index [35], providing an estimate of the stability of proteins. Most HvGRAS proteins were unstable with an instability index greater than 40. Eight HvGRAS proteins were probably stable with the instability of index from 25.76 to 39.77. Aliphatic index was described as the domination of aliphatic side chains in protein volume, highly aliphatic side indicated the thermal stability [35]. The aliphatic index of all HvGRAS proteins on average was 83.37, ranging from 60.28 to 101.08.

### 3.2. Phylogenetic Analysis of GRAS Proteins in Barley, Arabidopsis, Rice, Maize, Wheat and Brachypodium Distachyon

**Figure 1:**
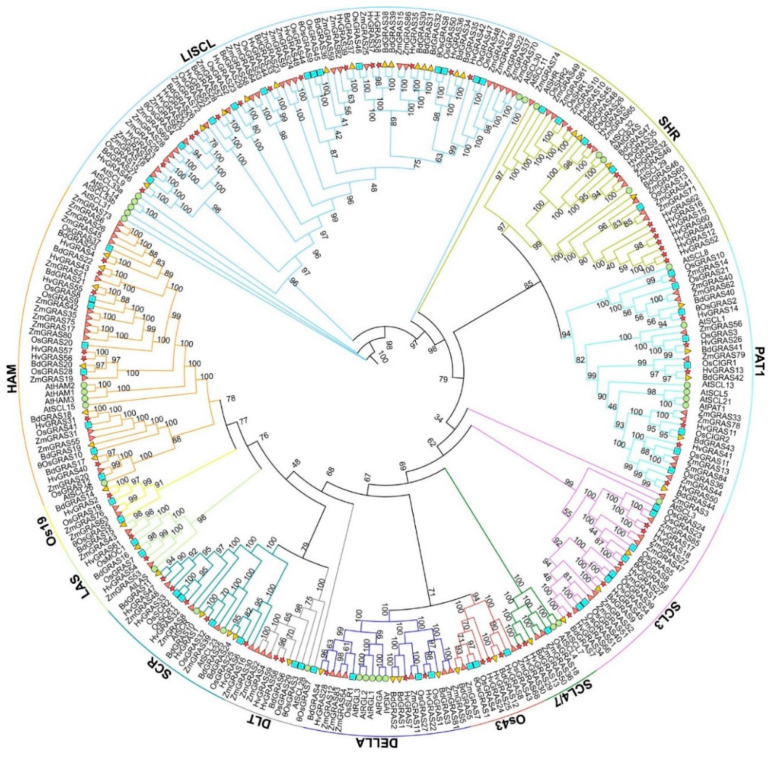
An unrooted phylogenetic tree of GRAS transcription factors. The phylogenetic tree (Maximum Likelihood) of GRAS proteins including Arabidopsis (green), rice (blue), maize (orange), barley (red), and *Brachypodium distachyon* (yellow) were generated. “θ” indicates for the pseudogene fragments.

To obtain clues about the evolutionary history of GRAS family proteins, the GRAS domain of 34 Arabidopsis, 60 rice, 86 maize, 179 wheat, 48 *Brachypodium distachyon*, and 62 barley GRAS were aligned with MUSCLE, and the results were used to generate phylogenetic trees using Maximum Likelihood method in W-IQ-TREE [37]. According to both the two clusterings and the relationship with known Arabidopsis, maize, rice and Brachypodium orthologs, barley GRAS proteins were clustered into 12 subfamilies including SCL3 (five members), Os43 (three members), DELLA (three members), SCR (five members), HAM (seven members), Os19 (one member), SCL4/7 (two members), LAS (two members), PAT1 (six members), SHR (10 members), LISCL (14 members), and DLT (two members). The features of each subfamily will be discussed later with their protein motifs (Section 3.4). We constructed two trees with or without wheat GRAS. The evolution relationships of the barley GRAS were identical, except that *HvGRAS48* together with two rice pseudogenes and two unknown function maize genes were grouped out of LISCL subfamily if wheat is included (Figure 1 and Figure S5). This can be interpreted as proteins in the overexpanded LISCL in the hexaploid share less conservation with HvGRAS48 than the other barley LISCL orthologs. Considering HvGRAS48 shares same motif arrangement with other barley LISCL (Section 3.4), we carried further analysis based on the tree without wheat. Similar to the GRAS family analysis in Arabidopsis and rice, the majority of HvGRAS mainly enriched in LISCL, SHR, SCR, PAT1, and HAM subfamilies. SCL3, Os43, DELLA, Os19, SCL4/7, LAS, and DLT comprised a few members [9]. Besides, subgroups lacking homolog to Arabidopsis were found, which was grouped with rice specific-protein namely Os43 and Os19 [10]. Strikingly, SCL3 subfamily consisted of only one Arabidopsis protein (AtSCL3). This similar phenomenon was also observed in DLT subfamily, where AtSCL28 separated individually from rice, maize, Brachypodium, and barley (Figure 1). The result suggests the divergent evolution of monocot or the process of gene loss during the duplication process. The phylogenetic tree indicated the random distribution of GRAS proteins in these five analyzed species. Take SHR subfamily as an example, Arabidopsis has four members, rice has five members, and Brachypodium has four members, while barley and maize consisted of 10 members (Figure 1).

Based on the phylogenetic tree results, distant relationship and classification suggested that the monocot specific Os43 group and Os19 group were likely divergent from SCL3 and HAM groups, respectively, during the evolutionary divergence between monocot and dicot (Figure 1). In each group, the hexaploidy wheat has roughly as three times members as the diploid counterparts (maize, rice, barley and Brachypodium). These proteins functions of Os43 group and Os19 group might have a monocot specific role. Not surprisingly, barley GRAS proteins generally share higher similarity with their orthologs in cereals (wheat and Brachypodium) than that in rice, maize, and Arabidopsis (Figure 1 and Figure S5) species relationships. The exceptions otherwise indicate uneven diversification rates of these orthologs. HvGRAS61, HvGRAS32, HvGRAS33, HvGRAS48, HvGRAS41, and HvGRAS42 outgroup with their orthologs in other species, indicating these barley proteins have more specific diversification from their common ancestors than in other species. More strikingly, a cluster of barley paralogs HvGRAS62/HvGRAS16/HvGRAS15/HvGRAS60/HvGRAS49/HvGRAS12/HvGRAS52 show less conservation than their counterparts in the same cluster. It appears that the ancestor gene diversified and duplicated in barley. It is also evident that these paralogs were formed after the divergence of the three cereal plants: HvGRAS17/HvGRAS18, HvGRAS29/HvGRAS30, HvGRAS37/HvGRAS38, HvGRAS56/HvGRAS57, and HvGRAS58/HvGRAS59.

### 3.3. Chromosomal Location and Evolutionary Analysis of HvGRAS Genes

**Figure 2:**
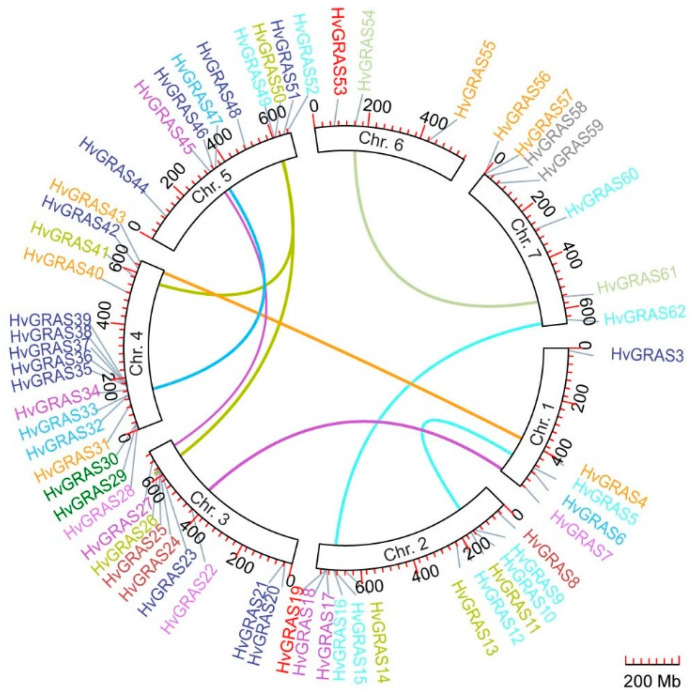
Chromosomal localization of HvGRAS genes and gene duplications. The locations of HvGRAS genes are based on the physical position. The black letters indicate chromosome number. Colorful lines and neighboring HvGRAS genes in same color indicate duplication.

All 62 *HvGRAS* genes were mapped on barley chromosomes, except two chromosome P1-derived artificial chromosomal localization unmapped genes: *HvGRAS1* (Accession No.: HORVU0Hr1G003230) and *HvGRAS2* (Accession No.: HORVU0Hr1G004640). The corresponding PAC (P1-derived artificial chromosome) consisting their coding sequences (PAC Accession No.: 38339934 and 38343134 respectively) are yet unmapped on the Hi-C (high-throughput/resolution chromosome conformation capture) map [32]. Generally, most *HvGRAS* genes were unevenly distributed on chromosome 2 and 4, with 12 and 16 genes. By contrast, chromosome 6 only consisted of 3 *HvGRAS* genes (Figure 2). The rest of the *HvGRAS* genes were located dispersedly on other chromosomes 1, 3, 5, and 7, but it was mainly positioned on the distal ends of the chromosome arm (Figure 2).

Duplication events were dominantly forced to the expansion of GRAS family during the evolutionary history. The phylogenetic analysis results identified 17 homologous pairs, which based on evolutionary relationship and distance between the homolog gene pairs (Figure 2 and Figure S3). Seven and 10 pairs of genes were predicted to undergo tandem and segmental duplication, respectively (Figure 2) [33]. The same subfamily genes located within 30 kb or neighboring intergenic region were registered as the tandem duplication [52]. Segmental duplication genes were determined based on duplication of genomic segment on different chromosomal location [52]. Supporting the results in many *HvGRAS* homology pairs were identified among the chromosome. The result indicated that both tandem and segmental duplication possibly contributed to the expansion of the subfamilies in barley. For instance, *HvGRAS17* and *HvGRAS18* in SCL3 could probably under the process of tandem duplication, whereas *HvGRAS27* and *HvGRAS45* within the same family were certainly the results from segmental duplication (Figure 2 and Table S2). Tandem duplication was probably observed in SCR group between *HvGRAS33* and *HvGRAS32*; meanwhile, *HvGRAS47* may be a segmental counterpart of these two genes.

To further explore the pressure of selective process in barley, synonymous (Ks) and nonsynonymous (Ka) nucleotide substitution rate of 17 homolog pairs was calculated (Table S2). Our results showed that 88.2% (15/17 *GRAS* pairs genes) had the ratio of Ka/Ks below 1, suggesting a purified selection proceed in barley *GRAS* genes.

### 3.4. Protein Motif and Gene Structure Analysis of HvGRAS Family

To investigate the GRAS domain in barley, multiple peptide sequence alignment was performed. Generally, most HvGRAS proteins consisted of more than two highly conserved domain regions: LHRI, VHIID, LHRI, PFYRE, and SAW (Figure S1). However, all GRAS domains were absent in HvGRAS57 proteins, and only a partial VHIID domain was existent (Figure S1). Likewise, almost GRAS domain was completely absent in HvGRAS48, except for the partial part of LHRI. However, numerous domains in HvGRAS48 were observed in the N-terminal region, suggesting that the potential functions of this protein which was different from other HvGRAS.

**Figure 3:**
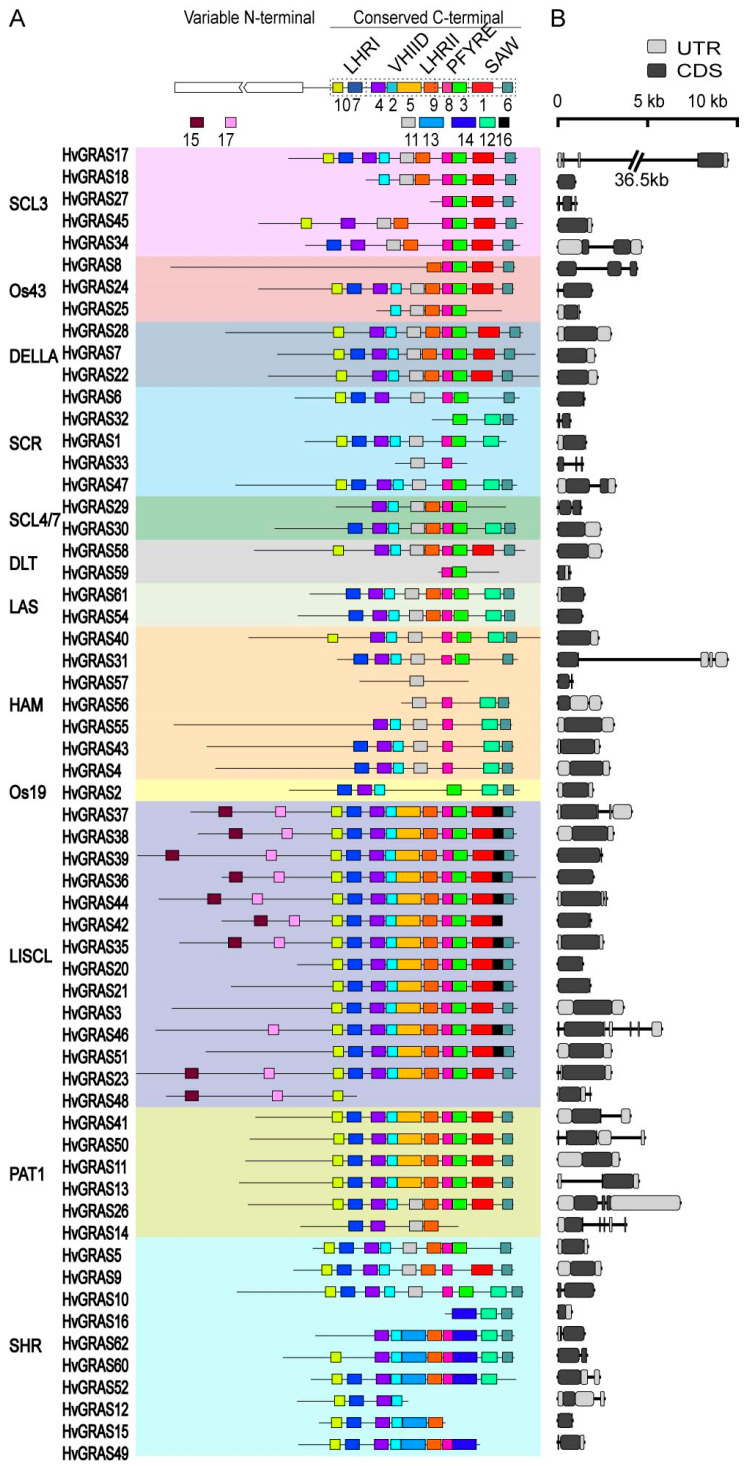
The structure of HvGRAS proteins and genes in barley. GRAS structure contains variable region in N-terminus and conserved region in C-terminus, numbers indicate the motif relevant to the domain. (**A**) The motif logo distribution of HvGRAS was generated by MEME. Each categorized motif logo was displayed in different box colors. (**B**) Gene structure of HvGRAS family genes in barley, UTR is represented by gray color, CDS region is marked in black color.

To further explore the characteristic of conserved domains in barley, 62 HvGRAS proteins were subjected to an online tool MEME [40]. Briefly, a total of 17 motifs were identified in barley and assigned from motif 1 to motif 17 (Figure 3, Figures S1 and S2). Generally, the distribution and characteristics of conversed motif were similar in the same GRAS subfamilies, suggesting the probably functional conversation. Consistent with the previous report in GRAS domains characterization analysis, the arrangements of motifs were identified in the LHRI-VHIID-LHRII-PRYRE-SAW structure domain [1]. Motifs 10 and 7 belonged to LHRI; motif 4, motif 2, and motif 5 were located in VHIID; the N-terminal part of motif 9 was LHRII; and the remaining C-terminal part of motif 9 connected with motif 8 and motif 3 corresponds to domain PRYRE and motif 1 and motif 6 to SAW domain (Figure 3). Certain domains were not found to form a structure, but it was still a part of the conserved structure domain [15]. If the rule was followed the nomination of Pysh, Wysocka-Diller, Camilleri, Bouchez, Benfey, lacking certain characteristics domains or a few motifs in barley, was generally normal, except for PAT1 members. For instance, several HvGRAS proteins in SCL3, SCR, SCL4/7, DLT, HAM has truncated either motif 10 or 7, or fully missed both motifs.

The complete VHIID domain was detectable in LISCL and PAT1 subfamily. The motif components motif 4 and motif 2 were more highly conserved in other subfamilies than motif 5. For example, over 40 HvGRAS proteins had motif 4 and motif 2; and 17 proteins had motif 5. Generally, these incomplete VHIID domains were connected to either motif 11 or motif 13, which was specific to SHR subfamily. Motif 9, corresponding to LHRII, was absent in SCR, HAM, and Os19 subfamilies. Although motif 8 was the most conserved motif found among all the HvGRAS proteins (52/62), the complete PRYRE domain was only distributed in LISCL and PAT1 subfamilies. The rest of the subfamilies proteins were lacking in either motif 9 or motif 3. Within the SHR subfamily, the truncated PRYRE domain was connected to motif 14. Similarly, though the high occurrence of motif 6 in HvGRAS proteins, the complete SAW domain was only identified in SCL3, Os43, DELLA, DLT, PAT1, and SHR. Motif 6 in the rest proteins was connected with either motif 12 or motif 1 and motif16. Motif 1, motif 16, and motif 6 types of SAW domain were unique in the LISCL subfamily. The HAM subfamily proteins (HvGRAS40, HvGRAS55, HvGRAS43 and HvGRAS4) contain a plant conserved ILARLN hexapeptide in front of motif 7 (Figure S4) [53]. The corresponding coding sequences are the target site of barley *miR171* (*Hv-miR171a/b*: UGAUUGAGCCGU/[C]GCCAAUAUC) (Figure S4). Moreover, we found that motif 15 and motif 17 were specifically present in the N-terminal of LISCL subfamily proteins. The two motifs were probably related to transcriptional co-activation functions [2]. Therefore, we propose that the further discovery in GRAS proteins family possibly focuses to define the GRAS proteins by these shorter motifs and to characterize each motif function rather than the previous five domains. After all, they are not certainly conserved than previously thought. The interaction and forming of a heterodimer with cross subfamilies proteins were common in GRAS family, such as SHR and SCL3. The distinct motif composition among subfamilies may explain why this is necessary. In this study, we found different motif arrangement in both barley SHR and SCL3 subfamilies. It is interesting to examine the possible link between the two families in the future.

To evaluate the different evolutionary and functional diversification of subfamilies in GRAS proteins, the structural organization of exons, introns, and UTR was further investigated (Figure 3). The results revealed that most *HvGRAS* genes had no intron 74.2% (46 out of 62). Particularly, all genes in DELLA subfamily (*HvGRAS7*, *HvGRAS22*, and *HvGRAS28*), DLT subfamily (*HvGRAS58* and *HvGRAS59*), and LAS subfamily (*HvGRAS54* and *HvGRAS61*) were intronless. The abundant introns were observed in SHR, SCR subgroups (Figure 3, Table S1). Besides, *HvGRAS17* (SCL3 subfamily) had a particularly long intron which covered up to 36 kb. Generally, gene structures were not in accord among the same subfamily, suggesting diversification of gene family.

### 3.5. Expression Profile of HvGRAS Genes in Different Barley Tissue

**Figure 4:**
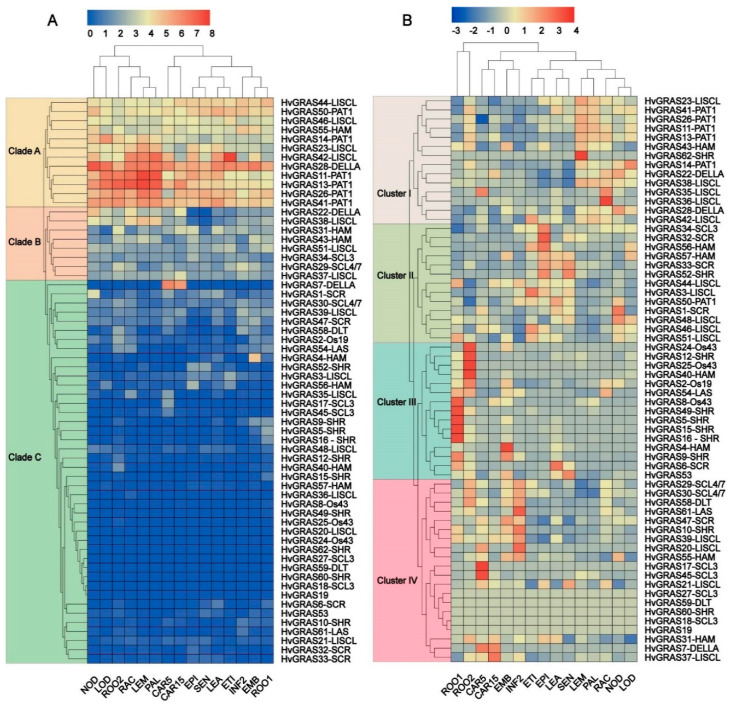
Gene expression profile of GRAS family member in barley (Morex cultivar) in different tissue. Expression profile of different tissue developing root, shoot, inflorescences and seedling stage were performed in barley. EMB, 4-day embryos; ROO1, roots from seedlings (10 cm shoot stage); LEA, shoots from seedlings (10 cm shoot stage); INF2, developing inflorescences (1–1.5 cm); NOD, developing tillers, 3rd internode (42 DAP); CAR5, developing grain (5 DAP); CAR15, developing grain (15 DAP); ETI, etiolated seedling, dark cond; LEM, inflorescences, lemma (42 DAP); LOD, inflorescences, lodicule (42 DAP); PAL, dissected inflorescences, palea (42 DAP); EPI, epidermal strips (28 DAP); RAC, inflorescences, rachis (35 DAP); SEN, senescing leaves (56 DAP). (10 DAP); ROO2, roots (28 DAP). (**A**) heat map is separated based on the high (Clade A), moderate (Clade B) and low (Clade C) expression. (**B**) heat map is performed by clusters row and column, Euclidean distribution method is applied to categorize similar expression pattern to one group (Cluster I to Cluster IV).

To preliminarily understand the functional role of *GRAS* genes in the barley developmental process, the expression pattern of *HvGRAS* genes in different tissues were retrieved from publicly available transcriptome data in BARLEX (Barley Genome explorer) [44]. The available transcriptome data were generated from the six-row Morex cultivar (one central spikelet and two fertile lateral spikelets). In general, the expression profiles of most *HvGRAS* genes were diverse among different subfamilies and expressed in at least one developmental stage. However, the expression of five *HvGRAS* genes (*HvGRAS18*, *HvGRAS19*, *HvGRAS27*, *HvGRAS59*, and *HvGRAS60*) were undetectable in the data set, and the expression pattern data of those genes were unavailable in the BARLEX, which is probably explained by the presence of uncharacterized pseudogenes. Hierarchical clustering following two normalization methods was adopted to cluster genes according to expression level (Figure 4A) and tissue specificity (Figure 4B). Based on the expression level information, *HvGRAS* genes were clustered as high, moderate and low expression (clade A, B, and C, respectively) (Figure 4A). Although a highly expressed gene could not be simply interpreted as physiologically more important than a lower expressed gene, and vice versa, these generally low expressed genes in clade C were likely not functional (Figure 4A). These genes are not only lowly expressed, but *HvGRAS18*, *HvGRAS19*, *HvGRAS27*, *HvGRAS59*, and *HvGRAS60* show no expression in any tissues (Figure 4A and Table S3), and also encode atypically short proteins that lack important domains (HvGRAS27 with 179 aa, HvGRAS32 with 156 aa, HvGRAS33 with 121 aa, HvGRAS59 with 126 aa, HvGRAS57 with 194 aa, HvGRAS16 with 142 aa, and HvGRAS53 with 61 aa) (Table S1). As shown in Figure 4B, the heat map was divided into four clusters according to the tissue specificity. Cluster I consisted of 14 members, in which PAT1 and LISCL subfamily were a dominantly high expression in various tissues such as inflorescence lemma, dissected inflorescences, inflorescences rachis, development tillers, and inflorescences, lodicule. Compare to other clusters, many *HvGRAS* genes in PAT1 and LISCL subfamilies were expressed specifically in these tissues, suggesting that these two subgroups participated in the regulatory of barley inflorescence formation (Figure 4B). Cluster II (13 members) had several genes belonged to LISCL, HAM, SCR, and SHR subfamily with the abundant expression in etiolated seedling, epidermal strips, shoots from seedlings, and senescing leaves. Generally, the similar expression pattern was identified in LISCL subgroup (*HvGRAS48*, *HvGRAS46*, *HvGRAS51*, and *HvGRAS3*), or HAM subgroup (*HvGRAS56* and *HvGRAS57*), (Figure 4B). Taking *HvGRAS56* and *HvGRAS57* genes as an example, *HvGRAS* genes, which may probably be a product of tandem duplication, also had a slightly similar the expression pattern (Figure 2 and Figure 4B). Fifteen members in Cluster III particularly expressed in roots from seedling development. The high expression of most *HvGRAS* genes in SHR subfamily was observed in root, indicated that the functionally conversed role of SHR subfamily in barley root formation. Interestingly, the new functional role of HAM and Os19 subfamily were explored in which *HvGRAS40* and *HvGRAS2* were also expressed abundantly in root respectively. Cluster IV consists of 20 members, numerous GRAS subfamily including SCL4/7, LAS, SCR, SHR, LISCL, HAM, and DELLA predominantly participate in embryos, inflorescence and grain development in barley. LISCL subfamily was also abundantly dominated in cluster IV, suggesting the imperative role in spike development. Rice (*OsMOC1*) and its putative ortholog in wheat controlled the number of spikelets per spike and panicle [25,26,27]. *HvGRAS54* and *HvGRAS61* orthologs with Arabidopsis (*AtLAS*), rice (*OsMOC1*) and maize, had a widen expression in various tissue (Figure 1 and Figure 4). Particularly, HvGRAS61 with high expression in spike was detectable, indicating the potential functions in barley inflorescence architecture (Figure 4).

### 3.6. Expression of 18 HvGRAS Genes during Barley Spike Development by RT-PCR

We conducted quantitative RT-PCR to further dissect the expression profiles of these inflorescence expression genes based on in silico analysis (above section). In this case, we used Golden Promise cultivar so that we could compare GRAS genes expression in the two-rowed Golden Promise and six-rowed Morex, where RNA-Seq data was generated, at the same time. We collected barley spike at different developmental stages: double ridge stage (DR), the lemma primordium stage (LP), the stamen primordium stage (SP), the awn primordium stage (AP), the white anther stage (WA), and the green anther (GrA), with two-week-old seedling as a control [54].

**Figure 5:**
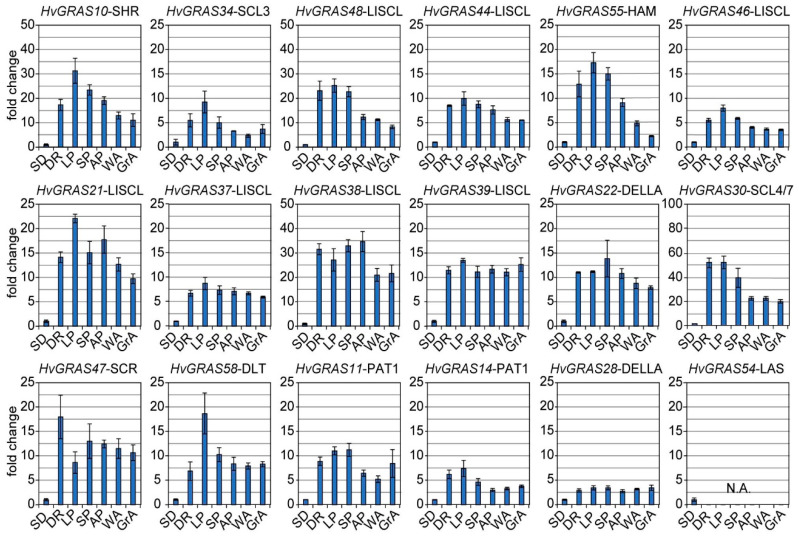
Relative transcript abundance of 18 genes in barley inflorescence (Golden Promise cultivar) developmental stage. qRT-PCR generates the RNA level in spike development including double-ridge (DR), lemma primordium (LP), stamen primordium (SP), awn primordium (AP), white anther (WA), green anther (GrA). Two-week-old seedling as a control. Bars indicate SD of three technical replicates.

Except for *HvGRAS54*, whose expression was not detectable (not as the RNA-Seq data suggested), most of our detected genes were highly expressed in developing inflorescence (Figure 5). Our data is largely consistent with the RNA-Seq data, demonstrating that the two-rowed barley and the six-rowed barley have no difference in GRAS genes expression. That also hints that these detected genes except *HvGRAS54* may not account for the row types difference between the two cultivars. LP stages peaked expressing genes include *HvGRAS10*, *HvGRAS34*, *HvGRAS48*, *HvGRAS44*, *HvGRAS55*, *HvGRAS46*, and *HvGRAS21*; *HvGRAS37*, *HvGRAS30*, and *HvGRAS58*; and *HvGRAS14* (Figure 5). There was a differential expression pattern in qRT-PCR result of *HvGRAS47* with highest expression at DR stages, suggesting that they could play a function of spikelet primordia initiation (Figure 5). Interestingly, our qRT-PCR analysis revealed that the canonical DELLA *HvGRAS28*, whose protein product consisted of a DELLA motif, was only moderately expressed, while the noncanonical counterparts *HvGRAS22* was abundantly expressed in spike developmental stages (Figure 5). This finding further demonstrated that this uncharacterized DELLA subfamily gene could probably control the barley inflorescence development.

### 3.7. Interaction Network of HvGRAS Spike Proteins

Forming a complex with other GRAS or other proteins is often required for GRAS proteins to exert their function. For instance, more than a dozen of direct DELLA interactors were reported, which explains the multifaceted role of DELLA in plant [55,56,57]. Interaction between two GRAS proteins are also common in this family, such as NSP1 and NSP2, SHR and SCR, etc. [21,22,23]. As 17 HvGRAS proteins are found highly expressed in developing spike (Figure 5), it is interesting to test their association and function dependence on each other. To this end, we conducted a barley protein interaction network based on the orthologs relationship with that in Arabidopsis, rice, and maize as the lack of sufficient evidence of protein-protein network in the large GRAS family in barley. A direct GRAS-GRAS interaction or indirect interaction though one common non-GRAS interactor were put in the network.

**Figure 6:**
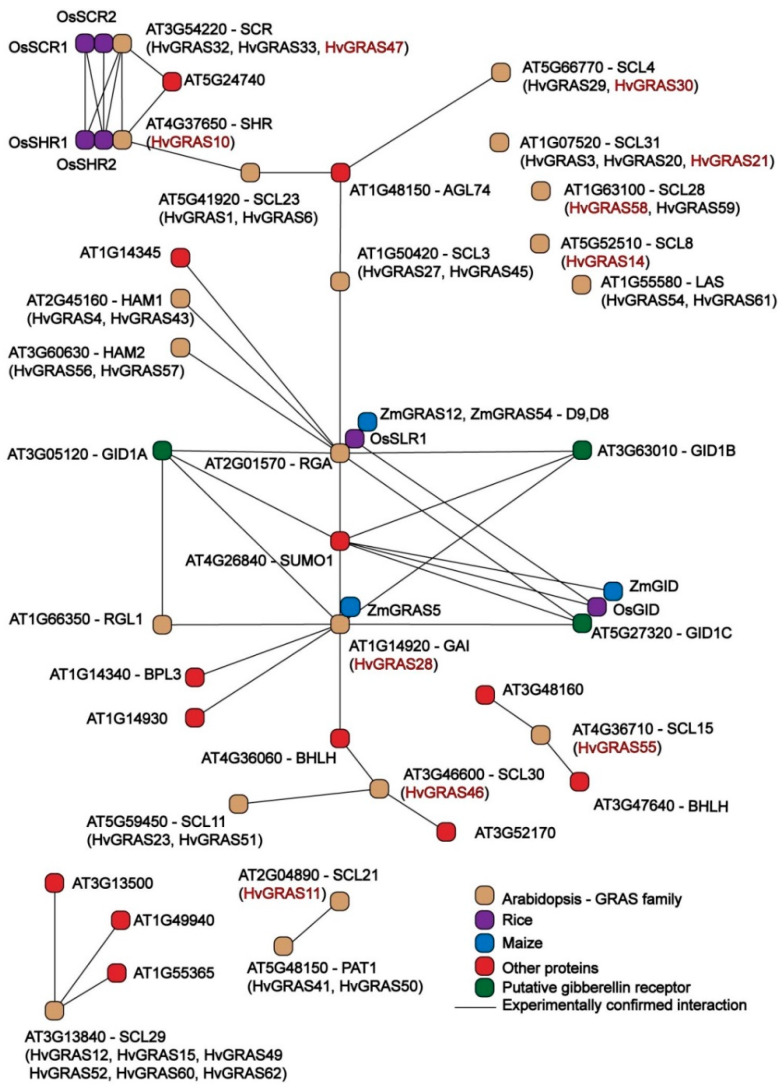
The potential interaction network of HvGRAS based on the Arabidopsis, rice, and maize orthologs. Black lines indicate the interaction, HvGRAS found expressed in developing spike are marked in red.

In general, 20 GRAS family protein in Arabidopsis, five in rice, three in maize, and 38 HvGRAS proteins orthologs were identified in the correlation network (Figure 6). Besides, the protein–protein interaction network of Arabidopsis SCL31, SCL28, SCL8, and LAS proteins were not found. Our correlation network prediction provided clue for studying the HvGRAS complexes in barley spike. The results indicated that barley DELLA proteins (HvGRAS28, HvGRAS7, and HvGRAS22) represent an interaction hub for connecting gibberellin receptor proteins, other HvGRAS proteins, and transcription factors (Figure 6). Besides, other proteins such as the bHLH family transcription factors may mediate interaction between HvGRAS (Figure 6). Our analysis also revealed that SHR-SCR-SCL23 module is another interacting hub (Figure 6). The two hubs are connected by the SCL3-DELLA protein interaction. Besides, PAT1 subfamily proteins (HvGRAS41 and HvGRAS50) are likely interact with that in SCL21 (HvGRAS11). These predictions based on homology are helpful in investigating the function mechanism of barley HvGRAS proteins.

## 4. Discussion

*GRAS* is a transcription factor family controlling a board range of developmental processes and stress response in plant [58]. The study of *GRAS* genes mutation in wheat *Rht-1* and *Rht-2* alleles provided a paradigm for using genetic variation in this gene family for crop improvement. It is particularly interesting that the functions in regulating cell division and differentiation of some GRAS members are conserved in angiosperms and these functions are generally important for agronomy traits. Although conserved function of HAM in maintaining apical meristem niche was shown in Arabidopsis and *Petunia hybrida*, whether they function similarly in cereal is not unclear yet [59]. MicroRNA *miR171* directly targets *HAMs* mRNA to repress *HAM* expressions. Overexpression of *miR171* in both rice and barley displayed defects in floral transition and spikelet architecture, consisting of the finding in Arabidopsis [43,60]. This provided a piece of indirect evidence for the conservation of *HAM* as well as its upstream regulator *miR171* in cereals such as barley. We identified *HvGRAS40*, *HvGRAS55*, *HvGRAS43*, and *HvGRAS4* as the putative target of barley miR171. Direct evidence, however, is needed to show their function in meristem maintenance. In contrast, the function of *LAS/LAS/MOC1* in flowering development varies in species. The tomato *ls* mutant is characterized by the absence of petals, while *Arabidopsis las-4* mutant developed a complete whorl of petals [27]. In rice and wheat, *MOC1* regulates spikelet numbers in the panicle/spike [25,61]. It may be interesting to investigate how the orthologs of *LAS/LAS/MOC1* in barley control inflorescence development. Besides, GA is shown as a florigenic signal and regulator of row numbers in barley [62,63]. It is thus perceivable that GRAS proteins are important for GA signaling to regulate barley inflorescence architecture. Indeed, transcriptome data suggest barley inflorescence development may require multiple factors from the GRAS family [45]. Nevertheless, the detailed investigation of GRAS family proteins in regulating barley spike development is scarce.

In the current report, we identified 62 barley GRAS proteins, most of which share five main highly conversed domains, namely, LHRI, VHIID, LHRII, PFYRE, and SAW motif (Figure 3 and Figure S1). Previous studies showed these motifs could mediate protein-protein and protein-DNA interaction [64]. For example, a point mutation in the conserved LHRI region of NSP2 interrupted the formation of the NSP1-NSP2 complex, an interfamily heteropolymer important for nodulation signaling, and hence interfered with nodule development in *M. truncatula* [65]. Mutations of rice SCL7 in the conserved LHRII and PFYRE motifs abolished the DNA binding capability of the protein [64]. Our phylogeny analysis clustered the barley GRAS to 12 subfamilies: SCL3, Os43, DELLA, SCR, HAM, Os19, SCL4/7, LAS, PAT1, SHR, LISCL, and DLT (Figure 3). The previous phylogenetic clusterings of this family were in substantial agreement though some fine-tuning in different plant species, indicating an intensive diversification in *GRAS* genes in angiosperms [7,66]. Our dendrogram is consistent with a phylogenetic analysis of GRAS proteins from a broad range of species including lycophyte and the bryophyte [9]. The GRAS protein numbers are similar with that in rice (57 *GRAS*), thought the barley genome (2n = 14, 5.1 Gb) is over 10 times bigger than that of rice (2n = 24, 430 Mb), explaining that barley genome consists abundant repetitive DNA region [33]. We identified two yet unknown function monocot specific GRAS subfamilies, Os43 and Os19, but no dicots unique GRAS subfamily. Nevertheless, the greater GRAS number comes from not so much in the advent of monocot specific protein families as greater gene duplication in barley. Tandem repeats of closely related *GRAS* homologs are commonly observed in chromosomal location diagram (Figure 2). Remarkably, seven close homologs (*HvGRAS35*-*HvGRAS39* genes belonging to the LISCL subfamily) are encompassed in a narrow 3 Mb region in chromosome 4. Besides, we identified 10 pairs of homologs arisen from segmental duplication across chromosomes, the pattern of which roughly resembles with the genome-wide synteny map in barley [67]. Like many other plant species [6,68], a large number of *GRAS* genes (74.2%, 46 out of 62) are intron free (Figure 3 and Table S1). Not surprisingly, the largest subfamilies (LISCL, SHR, HAM1 and PAT1) contain the most intronless genes (13, 8, 7, and 6, respectively). A big proportion of intronless genes are also common in other big gene families [69]. Generally, the intronless genes are likely resulted from horizontal gene transfer from intronless ancient prokaryotes genes, duplication of existing intronless genes, or retroposition of intron-containing genes [70]. It is reported that at least part of *GRAS* genes are originated from the horizontal gene transfer from ancient prokaryote [4]. Nevertheless, it neither rules out the other two ways of intronless genes generation nor an alternative “introns-early” model, i.e., the intronless gene are younger than and generated from intron containing genes [71]. Collectively, our data suggest that the duplication events are the major mechanism contributing to the massive expansion of *GRAS* gene family members in different species.

The phylogeny together with the expression profile also provides a clue for exploring the protein functions. Arabidopsis DELLA proteins including GAI, RGA, RGL1, RGL2, and RGL3 are negative master growth regulators involved in GA signaling [56]. These proteins are characterized as a conserved D-E-L-L-A (amino acids sequence) domain. Barley SLN1 or HvGRAS28 according to our nomination is the only barley protein to fulfill the criteria. It was reported to be regulated by GA in protein level, a conserved GA-DELLA module regulating plant height [55,72,73]. Following the GRAS analysis in Arabidopsis, rice, and Populus, we also classify HvGRAS7 and HvGRAS22, which share similarity with the known DELLA but without DELLA motif, into the DELLA subfamily [6]. The DELLA subfamily proteins form an interacting hub with many proteins (Figure 6). Our data revealed surprisingly *HvGRAS7* and *HvGRAS22*, but not the canonical DELLA *HvGRAS28* highly expressed in inflorescence development (Figure 5), where gibberellin has been shown as an important regulating role [63]. It is yet possible that a low-level expression of the DELLA protein in spike is enough for the gibberellin signaling. Otherwise, HvGRAS7 and HvGRAS22 have overtaken part of the DELLA role in spike perhaps in a different way with DELLA since DELLA motif has been shown important for gibberellin mediated DELLA degradation [74]. Five *HvGRAS* genes were clustered as SCL3 subfamily, a subfamily closely related to DELLA. Arabidopsis SCL3 interacts with DELLA and its expression is regulated by DELLA [17]. The LHRI-VHIID-LHRII region is important for the interaction [21]. For instance, the expression of HvGRAS18 and HvGRAS27 lacking this region are undetectable (Figure 3 and Figure 4), thus are not likely functional in barley. In the SCL3 subfamily, HvGRAS34 is ubiquitously expressed, while HvGRAS17 and HvGRAS45 are developing grain specific suggesting their diverged functions. Though castor beans Os43 proteins are closely related to SCL3 subfamily, they appear to be non-functional as their expression levels are relatively low in every tissue in castor beans [66]. Consistent with the study in castor bean, the barley Os43 members are also low expressed (Figure 4), and their loss of function remains to be vindicated. In Arabidopsis, the SHR and SCR subfamily proteins act together in endodermis specification in vasculature tissues [21,75]. SHR is the only characterized and perhaps the only functional protein within the Arabidopsis SHR subfamily, while the others include a pseudogene and two very low expressed genes [6,8]. Noticeably, the T-DNA insertion of the two genes did not interrupt their expression, providing further evidence to indicate their loss of function [8]. The barley orthologs of Arabidopsis SHR is HvGRAS10, whose function may interact with both SCR and SCL23 subfamily proteins in barley. In Arabidopsis, the SHR-SCR-SCL23 module was originally found to regulate endodermis development in roots [21]. Recently, accumulating data suggested that this module also regulates endodermis equivalent tissues in leaves [76,77]. As suggested by the previous studies, this module is likely conserved in angiosperms including barley [21]. Combining the putative interaction and expression profile, our data pointed for the first time that *HvGRAS47* and *HvGRAS10* may work similarly as Arabidopsis SHR and SCR in roots and leaves (Figure 4, Figure 5 and Figure 6). Nevertheless, we did not find high expression of SCL23 orthologs (*HvGRAS1* and *HvGRAS6*), but SHR (*HvGRAS10*) and SCR (*HvGRAS47*) expression in developing spike, suggesting a difference between roots and leaves. On the other hand, the two SCR proteins, SCR and AtSCL23, are functionally related but diversified, forming different heterodimers with the mobile master regulator SHR [77]. Their interaction combination, location of the expression and cross-regulation of SHR, SCR and AtSCL23 generate a regulatory network in the endodermis development of Arabidopsis roots and shoots [76].The 10 SHR and 5 SCR identified in the barley are likely sharing function with SHR and SCR in Arabidopsis, whose functions in rice and Arabidopsis have been shown conserved [21]. Like the Arabidopsis SHR subfamily, nevertheless, the partial or fully gene loss of function in the even more expanded SHR and SCR subfamilies in barley need to be carefully examined in the future, as large proportion of them are expressed at low level (Figure 4). We identified 7 HAM subgroup GRAS protein in barley. The upstream regulator of HAM1 type GRAS, microRNA miR71 was shown to include a conserved function as in rice and Arabidopsis. It supports that barley HAM1 subfamily proteins, as well as their upstream micro RNA function, are conserved. We identified *HvGRAS40*, *HvGRAS55*, *HvGRAS43*, and *HvGRAS4* as the putative target of barley *miR171*. Direct evidence, however, is needed to show their function in meristem maintenance. Conceivably, the barley HAM1 proteins may participate in similar processes like HAM1/HAM2/HAM3 in maintaining meristem homeostasis but in different stages, as their expression patterns are dramatically different (Figure 4). Two barley GRAS were identified belonging LAS subgroup, whose function has been shown conversed in Arabidopsis, tomato, and rice [1,8,27]. The two barley LAS GRAS genes, *HvGRAS54* and *HvGRAS61*, probably arose from segmental duplication (Figure 2). They likely share the conserved function with LAS/LAS/MOC1 in regulating lateral organ initiation, such as tillering [25]. One Os19 family GRAS, *HvGRAS2* was identified in barley, whose rice homolog, *OsGRAS19*, participates in brassinosteroid signaling to regulate leaf morphology, leaf angle, stem and grain size [78,79]. Further evidence is needed to see whether *HvGRAS2* act similarly as *OsGRAS19* to controls traits through brassinosteroid signaling pathway, as HvGRAS2 is widely expressed in many tissues like OsGRAS19, DLT subfamily proteins are also reported participating in brassinosteroid signaling in rice and Arabidopsis [80,81]. The DLT is a direct target of GSK protein kinases, an important brassinosteroid signaling transductor [81]. Two tandemly arranged barley DLT, HvGRAS58 and HvGRAS59, were identified. However, *HvGRAS58* may be the only DLT1 equivalent, as *HvGRAS59* is likely a pseudogene as encoding a short protein missing multiple domains and not detected expression in every tissue (Figure 3). SCL4/7 subfamily is a phylogenetic neighbor of DLT1 subfamily. We found two tandem arranged genes, *HvGRAS29* and *HvGRAS30* belong to this family. Their expression especially *HvGRAS30* gene in early inflorescence suggesting their roles for barley spike development. PAT1 proteins in Arabidopsis involves in light signaling pathways, with the close homologs within the family physically interacting with each other [58,82]. The PAT1 proteins also participate in other processes such as stem cell regeneration after wound by interacting with ETHYLENE RESPONSE FACTOR115 [83]. We identified six PAT1 subfamily members in barley. Five of them are highly homologs each other, while HvGRAS14 orthologous with AtSCL8 contains only two of those conserved domains shared by other PAT1 (Figure 1 and Figure 3). AtSCL8 is also the same, which was even categorized out of the PAT1 group by another research [82]. Remarkably, all the barley PAT1 are ubiquitously highly expressed (Figure 4), in agreement with that in rice and Arabidopsis [82]. Interestingly, *HvGRAS11* and *HvGRAS14* were observed highly expressed in developing spike (Figure 5). It suggests a previously unidentified PAT1 function in barley reproductive organ. LISCL subfamily is the largest GRAS subfamily in many species. There are two conserved subfamily restricted motifs in the N terminal of LISCL subfamily proteins, which have many acidic amino acids flanking hydrophobic or aromatic residue repeats. The two motifs are responsible for transcription activation [84]. This big subfamily has undergone dramatic function diversification. They were shown to regulate a range of processes such as microsporogenesis, adventitious root formation, abiotic stress resistance, and mycorrhiza symbiosis [84,85,86,87,88]. Fourteen barley LISCL proteins were identified. Our phylogenetic analysis suggests the subgroup could be further divided into two clades (Figure 1). While one clade has Arabidopsis, maize, rice, and barley members, the other is monocot-specific. As discussed above, extensive tandem duplication and segmental duplication may be evident for the monocot specific barley LISCL (Figure 2). The analysis of quantitative RT-PCR suggests that LISCL subgroups, including *HvGRAS21*, *HvGRAS37*, *HvGRAS39*, *HvGRAS44*, and *HvGRAS46*, are specifically expressed in the barley inflorescence, peaking at LP stages (Figure 6). This LP peaking expression pattern of these barley genes gives indication that they may function in the spikelet primordia development, as the number of spikelet primordia reach the maximum at LP stage. Given the gene expression profile and potential role in lateral organ initiation [25,26,27], it will be interesting to study the function of these *HvGRAS* in the lateral organs including floral components within a spikelet. It is noteworthy that these GRAS peak the maximum expression earlier than the row-type determinators, *Six-rowed spike* (*VRS*) genes (*VRS1-5*), repress the lateral spikelet fertility to different extend at carpel and awn emergence in developing lateral spikelets [89].

## 5. Conclusions

Taken together, our work laid a foundation to further elucidate the function of the barley GRAS members and provides valuable information about the gene functions of *GRAS* family in the development of barley inflorescence.

## Supplementary Materials

The following are available online at https://www.mdpi.com/2073-4425/11/5/553/s1, Figure S1: sequence alignment of GRAS family proteins, the barley GRAS proteins sequences were aligned by using Jalview software of Clustal W and Clustal X version 2.0, Figure S2: conversed motif domain analyzed from barley protein by MEME software, Figure S3: phylogenetic analysis of HvGRAS proteins in barley, Figure S4: mRNA of barley HAM subfamily genes are targeted by micro RNA miR171. (A) Two mature miR171 sequences are processed from precursor Hv-pri-miR171a. Hv-pri-miR171a is the only miRNA precursor of miR171 family identified in barley. (B) A plant conserved ILARLN hexapeptide was identified from barley HAM subfamily GRAS proteins. (C) Schemes of Hv-miR171a and Hv-miR171b binding sites in CDSs of HAM genes in barley. Figure S5: phylogenetic analysis of GRAS genes family in Arabidopsis, rice, maize, barley, wheat, and *Brachypodium distachyon*. Table S1: The physical position and chemical characteristics HvGRAS, Table S2: Ka/Ks analysis of 17 paralogous pairs in barley, Table S3: *HvGRAS* FPKM value of different tissue, Table S4: *HvGRAS* FPKM value of inflorescences, Table S5: list of sequence primers used in this study, Table S6: the expression data of qRT-PCR in barley inflorescence, Table S7: List of GRAS genes in Arabidopsis, rice, maize, wheat, and *Brachypodium distachyon*.
